# From Ambition to Action: Barriers and Enablers for the Inclusion of Indigenous Peoples and Local Communities in the Kunming–Montreal Biodiversity Framework and the Role of Non‐State Bridge Organizations

**DOI:** 10.1002/advs.202523991

**Published:** 2026-07-16

**Authors:** Susan Enechojo Ogbe, Jinfeng Zhou, Linda Wong, Jiachen Gao

**Affiliations:** ^1^ World Academy of Sustainable Development (WAOSD) Limited Hong Kong China; ^2^ Department of Physiology Federal University Wukari 200Km katsina‐Ala Road Wukari Taraba Nigeria; ^3^ China Biodiversity Conservation and Green Development Foundation (CBCGDF) Beijing China

**Keywords:** Biodiversity Conservation Policy, COP16, Global Environmental Governance, Kunming–Montreal Global Biodiversity Framework (KM‐GBF), Nature‐Positive Outcomes

## Abstract

The Kunming–Montreal Global Biodiversity Framework (KMGBF), adopted in 2022, set forth ambitious targets to halt and reverse biodiversity loss by 2030. However, translating these commitments into actionable strategies has proven challenging due to several factors. These include insufficient financing of biodiversity initiatives, lack of capacity and institutional coordination at the national and subnational level, fragmented implementation of the framework, limited integration of biodiversity into sectoral policies (agriculture and infrastructure, etc.), and gaps in monitoring, reporting, and accounting mechanisms. Moreover, geopolitical tension and uneven commitments among parties have further slowed collective progress. The 16th Conference of the Parties (COP16) to the Convention on Biological Diversity (CBD) was aimed at addressing these challenges. This paper analyzes the key outcomes of COP16, focusing on resource mobilization, monitoring frameworks, and the role of Indigenous Peoples and Local Communities (IPLCs). Building on institutional experience from the China Biodiversity Conservation and Green Development Foundation (CBCGDF), this Perspective highlights the important role that accompanying organizations and civil society can play in strengthening biodiversity governance. By synthesizing lessons from COP16 and identifying practical pathways for improving implementation, monitoring, financing, and inclusive participation, the paper offers recommendations that not only support delivery of the Kunming–Montreal Global Biodiversity Framework (KMGBF) but also help inform policy discussions and implementation priorities in the lead‐up to COP17.

## Graphical abstract

Graphical abstract illustrating the transition from global biodiversity ambition to on‐the‐ground implementation of the Kunming–Montreal Global Biodiversity Framework after COP16, highlighting the roles of governments, non‐state actors, scientific knowledge systems, and resource mobilization in achieving transformative biodiversity outcomes.

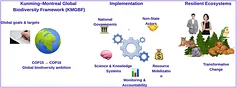

## 1 Introduction

The rapid decline of global biodiversity, driven by habitat destruction, climate change, pollution, and the unsustainable exploitation of natural resources, has triggered an urgent worldwide call for coordinated action [1]. In response, the global community adopted the Kunming–Montreal Global Biodiversity Framework (KMGBF) during the 15th meeting of the Conference of the Parties (COP15) to the Convention on Biological Diversity (CBD) in 2022 [2, 3]. Widely regarded as the “Paris Agreement for Nature,” the KMGBF marks a significant advancement in international biodiversity governance, setting a renewed agenda for halting and reversing ecological degradation [4].

Pivotal to the KMGBF lies a long‐term aspiration: to achieve “living in harmony with nature by 2050” [3]. This concept is enacted through four global goals and 23 action‐oriented targets to be completed by 2030 [5]. The framework is grounded in a theory of change that calls for systemic, cross‐sectoral efforts across all segments of society. It acknowledges that halting biodiversity loss is not only an environmental imperative but also a matter of social justice, sustainability, and resilience for present and future generations [6].

A key innovation of the KMGBF is the establishment of a monitoring framework intended to enhance transparency and accountability among member states. This mechanism enables nations to report on their progress using a set of domestically compiled indicators [7] while remaining sensitive to each country's unique data capacities and institutional contexts [8]. The framework addresses critical implementation shortcomings and monitoring challenges that hindered the success of the earlier Aichi Biodiversity Targets, and it aims to ensure more effective tracking of biodiversity commitments moving forward [9, 10].

The success of the monitoring framework hinges on three key factors: first, how comprehensively the indicators reflect the full breadth of the KMGBF's goals and targets; second, the degree to which countries adopt and integrate the framework to enhance their national monitoring systems; and third, the openness of data and metadata sharing [6]. When applied consistently, this system has the potential to catalyze adaptive policymaking at the national level and bolster global biodiversity governance.

In addition, the KMGBF is supported by a bold commitment to secure $200 billion per year by 2030 to advance biodiversity conservation efforts [3]. If fulfilled, this pledge could significantly strengthen environmental governance worldwide, particularly empowering countries in the Global South to pursue large‐scale habitat restoration, biodiversity protection, and sustainable development.

Nonetheless, putting this framework into practice has encountered several procedural hurdles. The initial COP16 session in Cali, Colombia (2024) [11], ended without consensus on various core issues, prompting a follow‐up meeting in Rome in February 2025 (CBD, 2025). The decisions made at the Rome session are expected to play a crucial role in defining how the KMGBF will be implemented.

As highlighted in recent global assessments, the pressures on biodiversity and ecosystem services are intensifying, bringing ecosystems closer to critical tipping points that threaten food systems, climate resilience, and public health [12, 13]. For the KMGBF to succeed, it must be inclusive, well‐resourced, and grounded in both scientific expertise and indigenous knowledge systems [14]. Effective implementation, broad participation, and strong international cooperation will be essential to achieving transformative outcomes [4, 14].

Furthermore, effective implementation of the KMGBF will depend not only on strategies, regulations, action plans, and financial commitments from actors of national governments [15], but also on the meaningful inclusion of Indigenous Peoples and Local Communities (IPLCs) whose stewardship practices and traditional knowledge form an essential pillar of global biodiversity governance [16, 17, 18]. Recent discussions, particularly those at COP16, have underscored the need to bridge long‐standing gaps between political ambition and grounded realities, leading to proposals for strengthened participation frameworks, new programs of work under Article 8(j), and more equitable benefit‐sharing arrangements, including emerging mechanisms such as the Cali Fund for Digital Sequence Information [19, 20, 21]. These developments highlight that enforceable safeguards must accompany commitments to inclusivity, legal recognition of Indigenous rights, and sustained capacity‐building if they are to translate into meaningful action on the ground [19, 20].

In parallel, COP16 marked a turning point in public engagement, widely described as the “People's COP” due to unprecedented participation from civil society, youth groups, academic networks, and community organizations, over one million participants across formal and informal spaces [24]. This surge in civic mobilization revealed a growing recognition that state‐led processes alone cannot deliver the transformative change envisioned by the KMGBF [25]. Instead, implementation will increasingly rely on distributed governance models that harness bottom‐up innovation, citizen science, community‐based monitoring, and environmental advocacy. Drawing on experiences from organizations such as the China Biodiversity Conservation and Green Development Foundation (CBCGDF), recent reflections show how civil society actors are operating within institutional gaps to catalyze legal innovation, defend ecologically sensitive areas, elevate traditional knowledge systems, and expand youth leadership in conservation [26]. These emergent people‐centered approaches signal a broader cultural shift in biodiversity governance, one that positions local actors not as peripheral stakeholders, but as essential co‐architects of the KMGBF's success. Although this Perspective focuses on the outcomes and implementation implications of COP16, it is equally intended to contribute to the next phase of global biodiversity governance by identifying priorities that may help shape discussions, decisions, and implementation efforts leading into COP17.

## 2 Resource Mobilization: Bridging the Funding Divide

Resource mobilization has been widely recognized as a crucial factor influencing the limited progress toward the Aichi Targets by 2020 [14]. In response, the KMGBF has attempted to address this shortfall by establishing three financial benchmarks, including the annual mobilization of USD 700 billion by 2030, as stipulated under Goal D, the redirection of USD 500 billion in incentives harmful to biodiversity (Target 18), and the increase in overseas development assistance from developed to developing countries to USD 20 billion by 2025 and USD 30 billion by 2030 (Target 19a) [27]. When compared with the estimated USD 130 billion invested globally in biodiversity in 2019 and the USD 4–10 billionfrom official development assistance (ODA) that year, the KMGBF financial commitments constitute a major escalation in both scale and ambition, reflecting the growing recognition that existing investment levels are insufficient in to achieve the Framework's 2030 implementation and close the global biodiversity financing gaps [28].

Financing biodiversity conservation remains a key area of discussion under the Convention on Biological Diversity (CBD), with ongoing efforts to scale up both the quantity and effectiveness of funding. While notable progress has been made through mechanisms such as the Global Environment Facility and various innovative financing initiatives, challenges persist in ensuring predictable, accessible, and equitable resource mobilization. Differences in perspectives between developed and developing countries regarding financing responsibilities and modalities continue to shape these negotiations, underscoring the need for collaborative solutions that build on existing successes [22]. Many developing countries have expressed interest in exploring additional or complementary financing arrangements beyond the Global Environment Facility (GEF) to enhance the stability and predictability of support. At the same time, several developed countries emphasize the value of continuing to use the GEF as the primary channel, noting its established governance structures and experience in managing biodiversity finance. These differing perspectives reflect the broader discussion on how best to expand financial flows while maintaining coherence, efficiency, and accountability within the global funding landscape [29].

The Multilateral Fund (MLF) of the Montreal Protocol is considered a good example of GBF funding needs because its dedicated financial and technical assistance enabled developing countries to effectively meet global environmental obligations [30]. Parties agreed to collectively mobilize at least USD 200 billion annually by 2030 to meet the targets set under the Kunming–Montreal Global Biodiversity Framework. Developed countries are committed to contributing USD 20 billion per year by 2025, increasing to USD 30 billion by 2030 [5]. The strategy integrates a diverse array of funding sources, including public and private investments, multilateral development banks, and innovative financial mechanisms [19]. The Global Biodiversity Framework Fund (GBFF), launched under the GEF, received initial contributions of approximately USD 382 million, signaling growing engagement, especially from private sector actors [31].

Nevertheless, the Global Environment Facility (GEF) continues to serve as a foundational pillar of global biodiversity financing and has mobilized substantial resources through its trust‐fund operations. At the same time, certain institutional characteristics of the GEF have been identified as constraining its full alignment with the Kunming–Montreal Global Biodiversity Framework (KMGBF). For example, although the GEF has disbursed billions of dollars for biodiversity conservation across four replenishment cycles since GEF‐5, the pace of project approvals and fund flows has occasionally been slower than desired [29]. These challenges underscore the urgent need to reform the financial architecture of the CBD and align funding flows more closely with the KMGBF's ambitious goals. Moreover, because the GEF is an independent financial mechanism serving multiple conventions rather than a subsidiary body of the Convention on Biological Diversity (CBD), its governance priorities and implementation timelines do not always coincide seamlessly with those of the CBD [32]. These observations do not diminish the GEF's significance; instead, they underscore the need for continued institutional adaptation and closer alignment with the KMGBF's implementation needs.

A significant positive development has been the establishment of the Global Biodiversity Framework Fund (GBFF) under the GEF, designed specifically to accelerate the implementation of the KMGBF. According to recent reports, by mid‐2025, the GBFF had opened its second programming tranche of approximately USD 161.8 million, with multiple project preparation grants approved and an expanding pipeline of biodiversity projects in developing countries [30]. This progress demonstrates institutional responsiveness and growing momentum in biodiversity financing, reflecting lessons learned from earlier funding experiences.

The Kunming Biodiversity Fund (KBF) is a new financial mechanism launched in May 2024 by China to support the Kunming–Montreal Global Biodiversity Framework (KMGBF) implementation in developing countries, complementing existing funds like the GEF and GBFF. In addition to the Global Environment Facility and the Global Biodiversity Framework Fund, the Kunming Biodiversity Fund (KBF) represents another important channel for mobilizing resources in support of the KM‐GBF. Established during the Kunming Phase of COP15, the KBF is intended to complement existing mechanisms by supporting developing countries in implementing their biodiversity priorities. To enhance overall financial efficiency, it will be essential to continue strengthening the operation of these mechanisms, including improving the transparency and timeliness of approval and disbursement procedures, and ensuring that emergency biodiversity finance can be accessed when urgently needed [31, 33]. Supporting measures such as capacity‐building, knowledge‐sharing platforms, and honoring international commitments to financial assistance will remain critical. At the same time, enabling developing countries to adapt their National Biodiversity Strategies and Action Plans (NBSAPs) to reflect national priorities and scientific needs will help ensure that mobilized resources translate into targeted, impactful action [31].

The broader goal of mobilizing up to USD 700 billion annually by 2030 remains formidable [34]. The GEF and GBFF mechanisms are critical components, but on their own, cannot close the biodiversity financing gap. Bridging this divide requires not only additional financial commitments but also faster disbursement, improved access for developing countries, and enhanced accountability to ensure that funding is strategically aligned with national implementation pathways. Future efforts should therefore focus on streamlining approval and disbursement procedures, promoting transparency and co‐financing through public‐private partnerships, and ensuring that funding windows remain inclusive of countries with limited institutional capacity, including mechanisms that empower Indigenous Peoples and Local Communities [35].

Comparative lessons can be drawn from the Montreal Protocol, whose dedicated Multilateral Fund has played a pivotal role in supporting developing countries. By providing predictable financial resources, facilitating technology transfer, and offering technical assistance to meet phase‐out commitments, the mechanism ensured that countries with limited capacity were able to comply while advancing national development priorities. Although biodiversity conservation is inherently more complex, without the linear causality of ozone‐depleting substances and driven by diverse socio‐ecological factors, the success of the Montreal Protocol demonstrates how an autonomous, well‐resourced, and implementation‐focused mechanism can accelerate progress [36]. As a result, several scholars suggest that introducing a similar complementary mechanism under the CBD could help better meet developing countries’ needs alongside the GEF [29].

The GBFF, although still in its infancy, has begun to provide funding to biodiversity conservation projects. By January 2025, it had disbursed approximately USD 39.78 million to projects in Brazil, Mexico, and Gabon [37]. To ensure long‐term sustainability, experts have called for extending the GBFF's operational timeline beyond 2030 or transitioning it into a standalone, sustainably managed biodiversity fund [38]. China's Kunming Biodiversity Fund, meanwhile, has approved nine projects across fifteen countries in regions including Central and Eastern Europe, Asia‐Pacific, Africa, and Latin America. This initiative has entered a new phase of KMGBF implementation, aiming to promote equitable and diversified financing [39].

## 3 Monitoring Framework: Enhancing Accountability and Adaptive Implementation

One of the key outcomes of the resumed COP16 session in Rome was the finalization of the Planning, Monitoring, Reporting, and Review (PMRR) mechanism, which is critical to ensuring transparency, accountability, and adaptive management in the implementation of the Kunming–Montreal Global Biodiversity Framework (KMGBF) [40, 41]. The monitoring framework seeks to provide a robust and coherent structure for Parties to assess their progress against the Framework's 23 targets and four overarching goals. By establishing common global indicators and encouraging the development of complementary national indicators, the PMRR mechanism aims to ensure that implementation efforts are both measurable and comparable across diverse contexts [42].

A key strength of the monitoring framework is its emphasis on inclusivity. It explicitly calls for the integration of disaggregated data, particularly on gender, age, geography, and the rights of Indigenous Peoples and Local Communities (IPLCs). This approach is designed to recognize the differentiated impacts of biodiversity policies and ensure that marginalized and underrepresented groups are meaningfully involved in monitoring activities [42]. The inclusion of such disaggregated metrics aligns with the growing recognition that biodiversity outcomes are often inequitably distributed and that progress cannot be fully assessed without acknowledging social dimensions [22].

Furthermore, the PMRR process mandates periodic national reporting, peer review, and synthesis reports at the global level. These reports are intended to contribute to the ongoing Global Review process, with the Global Report providing periodic assessments of progress scheduled for 2030. This cyclical review structure is expected to facilitate evidence‐based policy refinement and adaptive implementation, which are critical for ensuring the mid‐term correction of trajectories that deviate from agreed biodiversity targets [19].

Despite these advancements, several challenges undermine the potential effectiveness of the monitoring framework. One major concern raised by stakeholders and observers is the voluntary nature of gender‐responsive indicators. While the KMGBF emphasizes the need for gender equality and the empowerment of women and girls, the lack of mandatory gender metrics risks relegating this goal to an aspirational status rather than an actionable policy [22]. The omission of enforceable obligations could lead to inconsistent application across national contexts, particularly in countries where data on gender collection and mainstreaming are still limited.

Additionally, the sustainability of community‐based monitoring systems remains in question due to financial constraints. While the framework supports the participation of IPLCs in biodiversity monitoring, the mechanisms to ensure sustained funding for grassroots initiatives are underdeveloped [41]. Many IPLC organizations operate with minimal external support and lack the technical infrastructure to collect, manage, and report data in line with CBD guidelines [42]. This creates a risk of tokenistic inclusion rather than meaningful engagement, especially in regions where community rights are contested or poorly recognized.

To overcome these hurdles, parties and supporting institutions must invest in capacity‐building programs, ensure direct access to financial resources for IPLCs, and institutionalize gender‐ and rights‐based approaches within national biodiversity strategies [43]. Moreover, partnerships with civil society organizations, academic institutions, and technology providers could play a crucial role in enhancing data collection tools, participatory monitoring protocols, and data transparency platforms.

The Monitoring Framework adopted at COP16 lays a critical foundation for tracking progress on the KMGBF. However, its long‐term success depends on whether Parties can operationalize it with rigor, ensure that financial and technical resources reach the grassroots level, and embed inclusivity into the fabric of biodiversity governance.

## 4 Bridging the Gap Between Political Will and Execution: Participation, Knowledge, and Fair Benefit‐Sharing

Although the Kunming–Montreal Global Biodiversity Framework (KMGBF) has been lauded for its inclusive language and ambitious goals, a significant disconnect remains between policy ambition and practical implementation, particularly regarding the roles, rights, and benefits of Indigenous Peoples and Local Communities (IPLCs). At COP16, efforts to close this gap led to key institutional reforms [17, 18]. The most principal among them was the establishment of a Permanent Subsidiary Body under Article 8(j), designed to elevate the perspectives of IPLCs and formally integrate their traditional knowledge systems into the operations of the Convention on Biological Diversity (CBD) [16]. This new body will guide the embedding of Indigenous ecological knowledge in policy, while upholding cultural integrity and safeguarding intellectual property rights.

In addition, a new program of work under Article 8(j) was adopted, further underscoring the vital role of IPLCs in conservation and ecosystem restoration. The program endorses biocultural approaches and supports customary governance systems, recognizing IPLCs as longstanding stewards of some of the most biologically rich areas on the planet [16]. This represents a growing global acknowledgment that achieving the KMGBF targets is only possible through genuine partnerships with Indigenous and local actors whose traditional territories often overlap with key biodiversity hotspots.

Deep‐rooted structural and procedural challenges continue to obstruct progress on these inclusive commitments. IPLCs often lack equitable access to financial support, capacity‐building opportunities, and decision‐making spaces. Despite formal acknowledgment of the importance of Free, Prior, and Informed Consent (FPIC), many countries still lack comprehensive legal frameworks that uphold Indigenous land rights and governance practices [22, 23]. In addition, there is a clear deficit in accountability mechanisms, particularly in the absence of enforceable safeguards to ensure inclusive governance processes are consistently applied.

A noteworthy policy innovation at COP16 was the introduction of the Cali Fund—a funding mechanism intended to promote fair benefit‐sharing from the use of Digital Sequence Information (DSI) derived from genetic resources. The fund is structured to receive voluntary contributions, especially from private sector users of DSI, and redistribute them to biodiversity‐rich countries and IPLCs [19, 20]. This reflects an attempt to create an equitable model of benefit‐sharing that aligns with the realities of the digital era, where physical resources are often replaced by database equivalents.

Despite its promise, the Cali Fund also raises several concerns. Because its financing relies on voluntary rather than binding contributions, questions remain about whether it can provide sufficient, reliable, and sustained funding. The lack of enforceable obligations could lead to selective participation by countries and corporations, thereby reinforcing existing imbalances in access and benefit‐sharing [21]. Furthermore, important aspects of the fund's governance remain unclear, especially regarding the inclusion of IPLC representatives in decision‐making and oversight processes.

**FIGURE 1:**
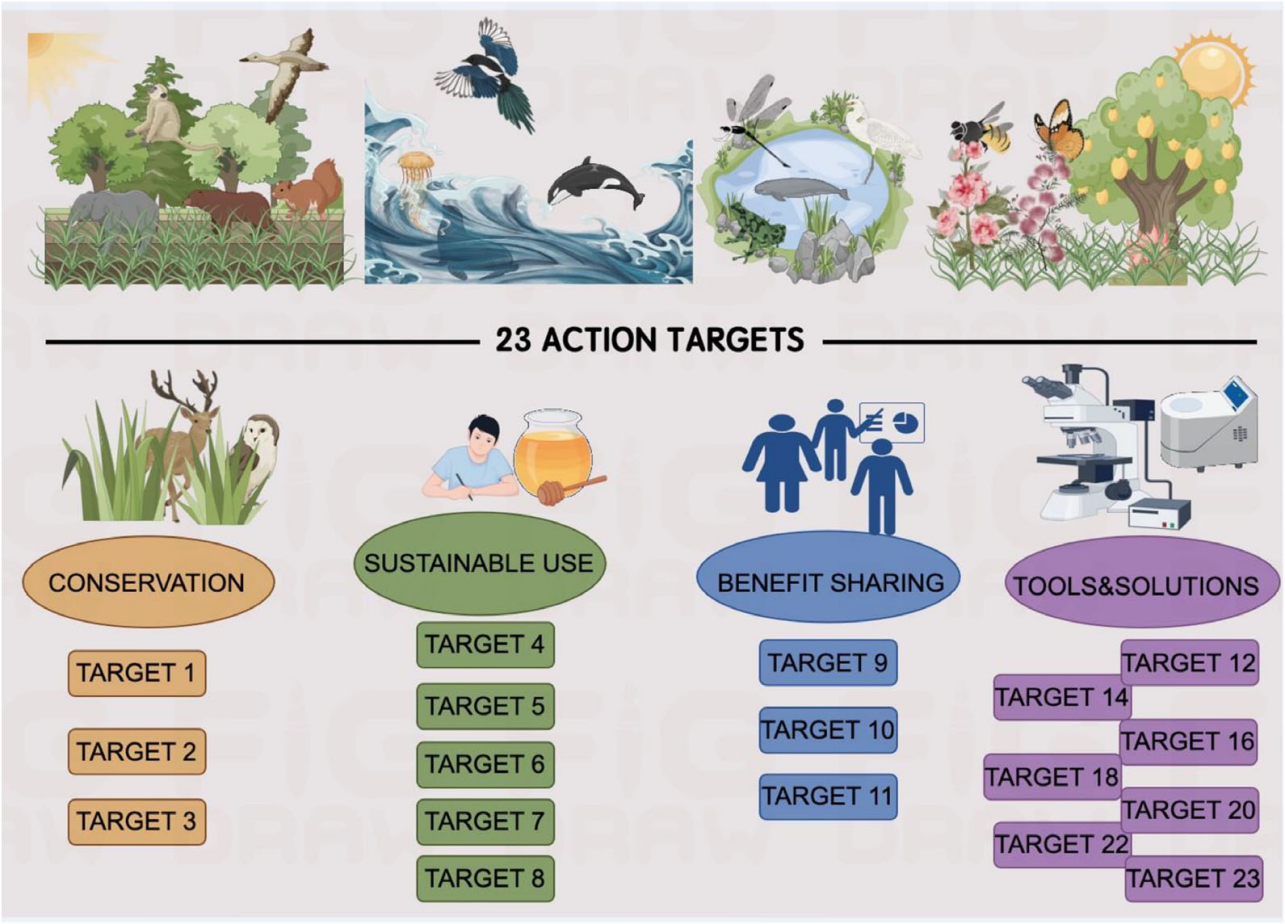
Structure of the Kunming–Montreal Global Biodiversity Framework (KMGBF). Illustrating the 23 action targets (in sectors: Conservation, sustainable use, benefit‐sharing, and tools & solutions).

These recent developments signal a broader shift toward recognizing the necessity of equity and inclusivity in biodiversity governance. However, unless they are matched by enforceable implementation tools, legal recognition, sustained financing, and strong institutional support, the gap between aspirational commitments and real‐world outcomes is likely to persist. Addressing this disconnect will require collective effort from Parties to the Convention, the private sector, and international funders, moving beyond rhetorical support to genuine, rights‐based engagement, where IPLCs are empowered as co‐leaders in conservation and sustainable resource management (Figure 1).

## 5 A Ray of Hope: COP16 as the People's COP

Amid ongoing implementation challenges, COP16 offered a rare glimmer of optimism. Dubbed 240 the “People's COP,” the conference marked a historic shift toward inclusive, participatory 241 biodiversity governance. With more than 23,000 officially registered delegates, including 242 government representatives, international organizations, Indigenous groups, civil society organizations, the private sector, and youth networks, and over 1 million people participating in activities within the Green Zone, COP16 became the largest and most publicly engaged CBD meeting in history. The meeting underscored a notable shift: biodiversity governance is no longer framed solely as a state‐centric responsibility but as a multi‐actor enterprise that depends on integrating scientific, local, and indigenous knowledge systems into decision‐making. This transition reflects the KMGBF's recognition that effective implementation requires broad societal participation, particularly from communities and organizations most directly engaged with ecosystems [17, 44].

A critical implication of this shift concerns the KMGBF's Planning, Monitoring, Reporting, and Review (PMRR) mechanism, which aims to improve transparency, enable adaptive learning, and support evidence‐based decision‐making at the national and global levels. Although the PMRR provides a structured architecture for monitoring progress toward the KMGBF's goals and targets, its current operational design remains heavily reliant on state‐reported information. As several assessments have noted, it has yet to fully integrate contributions from non‐state actors, including civil society, Indigenous Peoples and Local Communities (IPLCs), academic institutions, conservation NGOs, and philanthropic organizations [33, 45].

Recent analyses highlight that non‐state actors possess expansive monitoring capabilities, ranging from community‐generated biodiversity data to remote‐sensing platforms and citizen‐science networks, that could substantially enrich national reporting portfolios. China provides a representative example of how community monitoring and large‐scale biodiversity observation can enhance national biodiversity assessment. A basin‐wide study of bird diversity across the Yangtze River Basin, based on extensive monitoring data, revealed pronounced spatial and habitat‐specific trends, with increases in forest and farmland bird diversity in the upper and middle reaches and declines in wetland bird diversity in parts of the lower basin. These findings demonstrate the value of systematic monitoring and citizen‐science–supported datasets in informing conservation priorities and adaptive biodiversity governance [33, 44]. Yet these data streams are often episodic, unstandardized, or excluded from national reporting frameworks due to unclear mandates, limited governmental coordination, or a lack of formal recognition within the PMRR design. The absence of a coherent mechanism for data validation and integration further constrains the potential of these contributions to inform adaptive policymaking at both national and global scales.

The need for a more inclusive, multi‐evidence approach has been emphasized by scholars who argue that the KMGBF's success hinges on establishing governance systems capable of incorporating knowledge diversity in a manner that is scientifically credible and politically legitimate [45]. According to these authors, fostering structured participation from non‐state actors would help reduce information asymmetries, strengthen verification processes, and enhance the legitimacy of national reports, an outcome essential for avoiding the implementation pitfalls experienced during the Aichi Targets era.

This surge in civic involvement was not accidental. As early as COP14 and COP15, stakeholders— including our own delegation had proposed to the CBD Secretariat the idea of shaping future COPs into truly people‐centered platforms. These efforts bore fruit at COP16, where grassroots actors and public voices were given unprecedented visibility in shaping the biodiversity agenda [24].

This participatory transformation marks a critical moment in international biodiversity governance. In the face of insufficient governmental commitments and ongoing uncertainty around international funding mechanisms, especially given delays in operationalizing the Global Biodiversity Framework Fund (GBFF) and disagreements over digital sequence information (DSI)—the role of civil society, Indigenous Peoples and Local Communities (IPLCs), academic institutions, and youth networks has become more vital than ever [46, 47].

While progress on high‐level political commitments and binding financing frameworks remained uneven, COP16 reflected a growing societal consciousness around biodiversity and a mounting willingness from local actors to fill governance gaps. This dual reality, of state inertia on the one hand and civic dynamism on the other, signaled a possible paradigm shift: from centralized, state‐led negotiations to a more decentralized and distributed model of biodiversity governance [48].

In this evolving model, civil society, community organizations, academic institutions, and youth networks are no longer peripheral actors but are emerging as core agents of change. They play a vital role in bridging the distance between global targets and local realities through community‐based conservation, advocacy, knowledge exchange, and citizen science.

Thus, even as COP16 reaffirmed the structural weaknesses of international biodiversity governance, it also unveiled new spaces of possibility. These are spaces where bottom‐up leadership, collaborative innovation, and cross‐sectoral partnerships are beginning to redefine the boundaries of what is politically and ecologically feasible [5, 11].

This context highlights the importance of non‐state actors–particularly environmental foundations and civil society organizations–in advancing biodiversity conservation. As traditional governance structures often struggle to keep pace with ecological challenges, these organizations have emerged as a critical catalyst for change. Through legal activism, citizen science, youth engagement, and transboundary cooperation, they fill implementation gaps and experiment with innovative approaches that complement state‐led efforts. In the following section, we explore how China Biodiversity Conservation and Green Development Foundation (CBCGDF), as a representative example of how such organizations can operate within and around institutional constraints to achieve tangible conservation outcomes.

## 6 Cultivating Change in the Cracks: Barriers and Breakthroughs in People‐Centered Biodiversity Governance

In recent years, a growing number of organizations have emerged as pivotal actors in addressing complex sustainability challenges. Positioned at the intersection of state, market, and civil society, these institutions operate within institutional gaps left by traditional governance systems. Often motivated by a mission‐driven ethos, they function as intermediaries – translating scientific knowledge into policy, mobilizing grassroots action, facilitating cross‐sector collaborations, and holding power structures accountable. Their flexible, adaptive nature allows them to innovate where more rigid systems falter, making them especially effective in advancing biodiversity conservation and environmental justice.

The China Biodiversity Conservation and Green Development Foundation (CBCGDF) exemplifies this emerging category of “bridge organizations.” Established as a national public foundation dedicated to environmental protection and sustainable development, CBCGDF works across multiple domains, including public interest advocacy, community mobilization, citizen science, species conservation, and ecological culture promotion [49]. Over the past decade, it has become known domestically and internationally for promoting ecological civilization, supporting grassroots environmental defenders, coordinating nationwide volunteer networks, advancing biodiversity research, and participating actively in global biodiversity governance debates. As one of China's most active civil society institutions in this field, CBCGDF operates within the constraints of a highly centralized governance system while exploring innovative pathways for people‐centered environmental action. Its experiences provide valuable lessons on how organizations can catalyze meaningful change even in settings where civic participation is limited or politically sensitive [49, 50].

This section aims to deepen reflection on what limits people's participation in biodiversity governance and how our work demonstrates pathways to empower civil society, communities, youth, and academia. Although rooted in China's institutional and cultural context, the lessons from our experience have a broader relevance. Many countries, regardless of their political system, face comparable challenges in fostering environmentally inclusive governance, ranging from limited civic space to barriers in community engagement. Our work demonstrates how organizations can constructively work within existing systems to open new avenues for participation, collaboration, and impact. To structure this reflection, we present two parts: (a) persistent barriers to bottom‐up change, and (b) recommendations and real‐world illustrations of people‐driven interventions that CBCGDF has supported or led.

### 6.1 Breaking Barriers: Challenges to Inclusive and Impactful Participation

Despite growing rhetorical support for participatory biodiversity governance, numerous structural, legal, and ideological barriers persist across both the global South and North. CBCGDF's experience in policy advocacy, litigation, and community engagement has brought the following five categories of constraint into focus:

1. **Restricted Access to Policy Forums**: Civil society organizations, grassroots communities, and youth movements may face challenges in accessing policy‐making spaces in some contexts; however, there are also examples where these actors are actively engaged and contribute meaningfully to environmental governance. At both domestic and international levels, the lack of transparent processes, public consultation mechanisms, and civic representation hampers people's ability to shape conservation priorities [26, 51]. For instance, in previous COP meetings and national biodiversity strategy consultations, youth and Indigenous groups have been underrepresented or invited only in symbolic roles.
2. **Financial and Logistical Constraints**: Despite increasing recognition of the need to fund locally led action, only a fraction of international biodiversity finance reaches community‐level actors. According to a 2022 analysis by Rainforest Foundation Norway, less than 1% of global climate adaptation finance was directed to Indigenous peoples [52]. Conservation funding still favors large INGOs or multilateral agencies, often operating on short project cycles with rigid frameworks that exclude traditional knowledge or non‐Western metrics of success. This top‐heavy system perpetuates dependency and undermines long‐term ecological stewardship. For example, in West Africa, we have observed that donor‐driven coastal restoration programs often bypass local fishers’ associations, despite their generational knowledge of mangrove regeneration and fishing cycles. When these local systems are not resourced or respected, restoration efforts tend to collapse once external funding ends. As CBCGDF has seen while supporting grassroots biodiversity efforts across regions such as Yunnan, Inner Mongolia, and Hainan, groups working closest to ecosystems are often least equipped to engage with international donors, monitor biodiversity, or document violations due to resource limitations [53, 54].
3. **Knowledge and Narrative Gaps**: Formal conservation institutions often privilege scientific, Western‐style knowledge over Indigenous and community‐based knowledge systems. This epistemic bias marginalizes local experiences and spiritual values that are essential for stewardship. It also obscures the contributions of non‐academic actors. For instance, CBCGDF's work with community groups in northern China has revealed that many local plants, insects, and amphibians are routinely dismissed as “pests” or “weeds,” despite being keystone species in urban ecosystems [55]. Another example is that the indigenous fire management practices in Australia have proven more effective in reducing wildfire risks than high‐tech interventions, yet they continue to be overlooked in national climate‐biodiversity planning. Through biodiversity education walks, storytelling, and citizen‐science mapping, residents have begun reframing these biodiversities as ecological assets, illustrating how narrative shifts can foster public stewardship [56, 57].
4. **Institutional Rigidity and Technocratic Planning**: In many regions, conservation is still approached as an engineering or land‐use zoning challenge rather than a social and cultural process. This results in interventions such as the construction of concrete wetlands, artificial reefs, or greenwashing mega‐parks that prioritize aesthetics, tourism, or carbon metrics over biodiversity, ecosystem functioning, and community relationships. These tendencies are evident in several urban ecological restoration projects where engineering‐oriented interventions have prioritized landscape aesthetics over ecosystem integrity, highlighting the limitations of technocratic conservation approaches. Conservation planning processes remain dominated by technocratic models and top‐down interventions. Government and donor‐led projects often prioritize infrastructure and engineering solutions, sidelining community consent or participation. As CBCGDF's opposition to a wetland forest park in Beijing showed, such projects may destroy functional ecosystems to create aesthetically pleasing but ecologically empty simulations [53, 55].
5. **Legal and Civic Restrictions**: In many contexts, including China, the operational space for civil society organizations is shaped by legal frameworks that limit protest, advocacy, or independent environmental monitoring. Laws that restrict foreign funding for NGOs, impose onerous registration requirements, or broadly define “national security” concerns have been used to silence or dismantle environmental movements. For instance, in Southeast Asia and Central Africa, environmental defenders are frequently targeted for criminalization, intimidation, or violence—often in connection with industrial resource extraction or infrastructure development. The 2023 Global Witness report found that over 170 land and environmental defenders were killed in a single year, with Indigenous peoples disproportionately affected. This chilling effect on civil society undermines biodiversity efforts at their root. Where communities are unable to organize freely or speak truth to power, conservation becomes either tokenistic or top‐down. Legal systems that fail to protect collective land rights— especially for Indigenous and local communities‐ also erode the foundational custodianship that sustains biodiversity across over 30% of the Earth's surface. CBCGDF has experienced both breakthroughs and constraints while pursuing public interest environmental litigation, sometimes leading to major policy shifts, other times facing bureaucratic obstruction or political pushback [49]. While the specific legal and civic landscape varies across countries, the barriers described here are far from unique to China. Across civic society organizations engaged in environmental work often navigate complex political, legal, and institutional constraints.

### 6.2 Pathways for Empowering People: Reflections and Recommendations Towards Inclusive Biodiversity Governance

Accelerating the transition from ambition to action in biodiversity governance requires meaningful public engagement. Through experience, we have identified five strategic pathways that can empower civil society, foster inclusive participation, and bridge the gap between top‐down commitments and bottom‐up change [26, 58]. These approaches are not exclusive to any one region or system, but adaptable to a variety of governance and cultural contexts.

**FIGURE 2:**
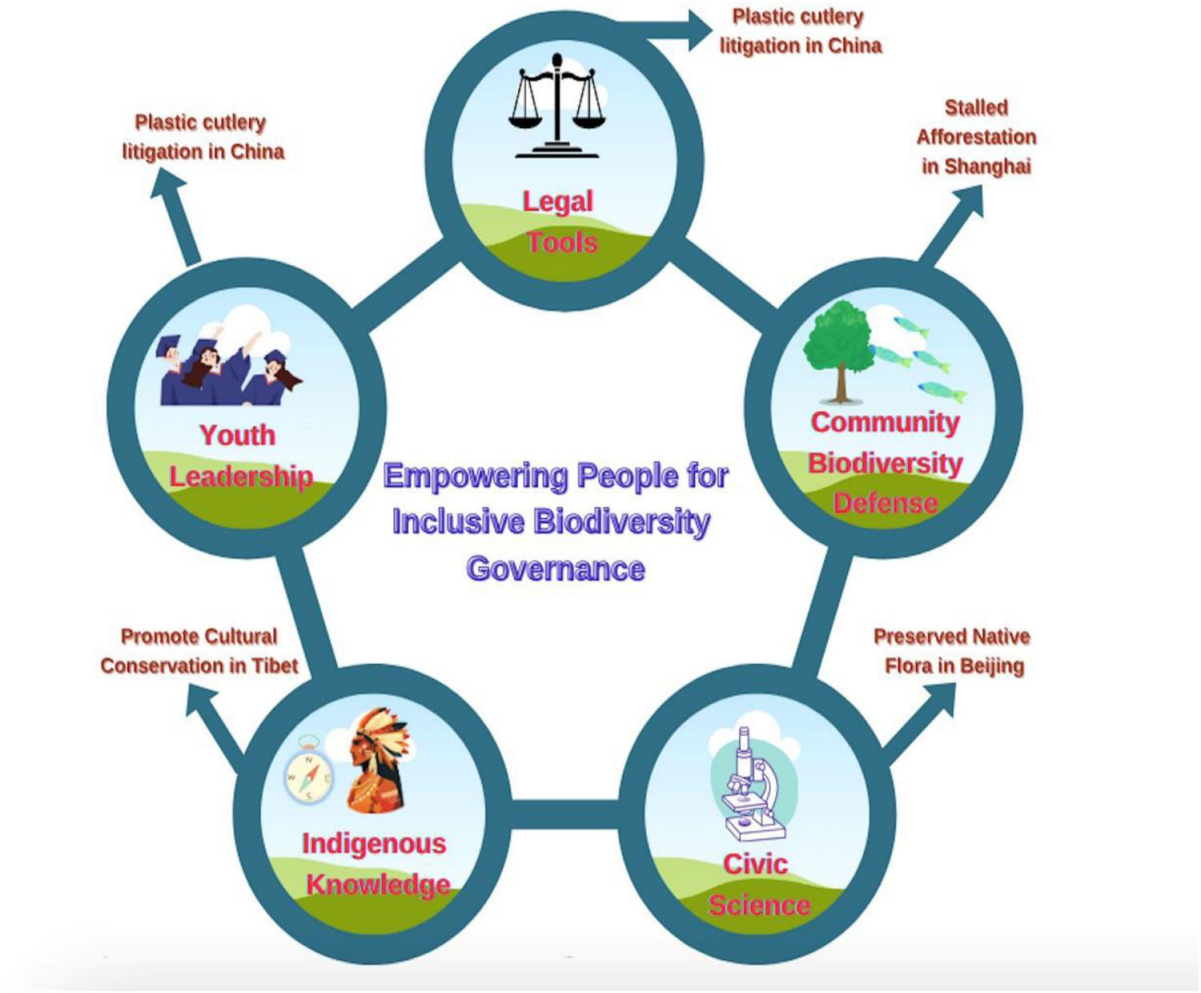
Five strategic pathways for empowering civil society and accelerating implementation of biodiversity goals. Each pathway is illustrated by real‐world applications, demonstrating how legal innovation, community engagement, civic science, Indigenous knowledge integration, and youth leadership can bridge the gap between topdown commitments and grassroots biodiversity action.

1. **Legal Tools to Shift Norms and Trigger Behavioral Change**: Legal action is not only a mechanism for top‐down regulation, but it can also be a grassroots tool to shift public norms and catalyze institutional responsiveness. When citizens are equipped with legal literacy and tools for redress, they actively shape environmental outcomes, often forcing policy adjustments even without formal power [59]. Example: In China, litigation against the automatic inclusion of plastic cutlery in food delivery–originally driven by citizens and environmental advocates–reshaped industry norms and prompted legislative reform [53]. The campaign mobilized delivery workers, consumers, and online platforms, showcasing how legal tools can catalyze bottom‐up behavioral shifts and pressure institutions to adopt sustainable practices.
2. **Centre Communities as Defenders of Local Biodiversity**: Conservation is most resilient when rooted in local stewardship. Community‐based action can prevent ecologically damaging projects and expose hidden impacts that centralized governments may overlook. By supporting locally led initiatives and amplifying community voices, organizations can co‐create solutions that are culturally and ecologically informed [58]. Example: In Shanghai's Nanhui wetlands, local alliances of birdwatchers, scientists, and concerned citizens opposed an afforestation project that planted non‐native species in sensitive intertidal zones. This grassroots mobilization brought media attention, policy review, and a shift away from inappropriate “green” engineering. Similarly, in Yunnan's freshwater ecosystems, river hardening projects that destroyed habitat under the guise of flood management were also opposed by community‐led biodiversity defense [55].
3. **Promote Civic Science to Build Knowledge and Accountability**: Citizen‐led monitoring enhances biodiversity data while fostering environmental literacy and shared ownership. Civic science initiatives help decentralize ecological knowledge, making it more actionable and legitimate in both public and policy domains [51]. Example: In Beijing, community patrol groups have mapped urban biodiversity, challenged overzealous landscaping practices, and called for the preservation of native flora, often dismissed as “weeds.” These volunteers do more than collect data—they reframe public perceptions, build civic pride in biodiversity, and create pressure for urban ecological reform [49, 54].
4. **Integrate Indigenous and Rural Knowledge into Public and Policy Dialogue**: Traditional knowledge systems are reservoirs of ecological insights and sustainable practice. Biodiversity governance can be enriched by respecting, documenting, and integrating these systems into broader conservation frameworks. Example: partnerships with ethnic minority communities in biodiversity‐rich areas, such as Tibet and Guizhou, have helped to promote cultural conservation, document traditional agricultural systems, medicinal plant knowledge, and sacred natural sites [49, 53]. By translating these practices into policy recommendations and public education campaigns, this has bridged grassroots traditions with national biodiversity strategies.
5. **Embed Youth Leadership as a Pillar of Biodiversity Action**: Young people are not just future stakeholders; they are present‐day leaders, innovators, and communicators. Institutionalizing youth roles within environmental organizations, education systems, and international forums ensures intergenerational equity and long‐term momentum [60]. Example: through youth‐led campaigns on wildlife trades, plastic pollution, and endangered species, as well as education initiatives like biodiversity‐themed Model UN simulations, leaders have driven awareness and policy shifts at both national and international levels [60, 61] (Figure 2).

## 7 Conclusions

The adoption of the Kunming–Montreal Global Biodiversity Framework marked an important political milestone, but its long‐term significance will ultimately depend on whether global commitments can be translated into effective, equitable, and accountable implementation. As this review has shown, progress will require more than increased financial resources or expanded monitoring systems. It will depend equally on governance arrangements that can integrate diverse knowledge systems, strengthen institutional accountability, and enabling meaningful participation by Indigenous Peoples and Local Communities, civil society, academia, youth, and other non‐state actors.

The analysis presented throughout this paper demonstrates that the challenges facing KMGBF implementation are deeply interconnected. Financing mechanisms must become more accessible and responsive to national priorities; monitoring frameworks must generate transparent and actionable evidence for adaptive governance; and implementation strategies must move beyond state‐centric approaches to recognize that durable biodiversity outcomes are shaped through collaboration across governments, scientific institutions, communities, and civil society. These dimensions are mutually reinforcing, and progress in one area is unlikely to be sustained without corresponding advances in the others.

The practical examples discussed in this review illustrate how people‐centered approaches can complement formal policy processes by strengthening public participation, facilitating knowledge exchange, supporting evidence‐based decision‐making, and improving accountability. Rather than representing isolated case studies, these experiences demonstrate broader governance principles that are transferable across different institutional and socio‐political contexts. They underscore the value of collaborative partnerships in bridging the persistent gap between global biodiversity commitments and implementation on the ground.

Ultimately, the success of the KMGBF should not be measured solely by the achievement of individual targets, but by its capacity to catalyze enduring institutional change and foster more inclusive models of biodiversity governance. Achieving the transformative vision of “living in harmony with nature” by 2050 will require sustained political commitment, adequate and equitable financing, robust monitoring systems, and the meaningful engagement of all sectors of society. Only by integrating these elements into coherent implementation pathways can the ambitions agreed at COP15 and advanced through COP16 be translated into lasting conservation outcomes. As the global biodiversity community prepares for COP17, these lessons provide a practical foundation for advancing implementation, strengthening accountability, and ensuring that future negotiations remain focused on translating political commitments into measurable conservation outcomes.

## Conflicts of Interest

The authors declare no conflicts of interest.

## Data Availability

The authors have nothing to report.
